# Revisiting the flexion-valgus type unicondylar posterolateral tibial plateau depression fracture pattern: classification and treatment

**DOI:** 10.1186/s13018-023-04318-y

**Published:** 2023-11-02

**Authors:** Bin Zhu, Jian Chen, Yu Zhang, Lijun Song, Jiahu Fang

**Affiliations:** Department of Orthopaedics, The First Affiliated Hospital of Nanjing Medical University, Nanjing, China

**Keywords:** Posterolateral tibial plateau fracture, Flexion-valgus force, Frosch approach, Posterolateral wall

## Abstract

**Objective:**

This study aimed to reclassify posterolateral tibial plateau fractures caused by a flexion-valgus force and describe this fracture pattern to provide a relatively programmed surgical treatment based on morphological characteristics that may improve reduction and stabilization.

**Methods:**

We retrospectively reviewed the fracture pattern and injury mechanism of patients with posterolateral tibial plateau fractures who underwent surgery at the First Affiliated Hospital of Nanjing Medical University between January 2014 and April 2020. The cohort was divided into three types. Type I was a depression fracture of the posterolateral platform with an intact posterolateral cortex. Type II was a depression fracture of the posterolateral platform with a disrupted posterolateral cortex. Type III was a depression fracture of the posterolateral platform in combination with anterior cruciate ligament (ACL) rupture or tibial insertion avulsion fracture of the ACL. The lateral window of the modified Frosch approach with an L-type locking plate was used for patients with type I and type III fractures. For patients with type II fractures, both lateral and posterolateral windows of the modified Frosch approach were used for surgery, and a T-plate on the posterior side with an L-plate on the lateral side were used for fixation. The Rasmussen radiology scoring was used to evaluate the quality of surgical reduction and the Rasmussen functional scoring evaluation standard was used to evaluate knee joint function.

**Results:**

A total of 69 tibial plateau fractures (36 male, 33 female) involving the posterolateral platform were discovered and included in this study. All patients suffered flexion-valgus force at the moment of the accident. There were 32 cases of Type I fracture, 28 cases of Type II fracture, and 9 cases of Type III fracture. The patients were followed up for 12–30 (mean 20.8 ± 9.4) months. The postoperative Rasmussen radiological scores for the three types of fractures were 15–17 (mean 16.31 ± 0.78), 14–17 (mean 15.93 ± 0.94), and 14–17 (mean 16.22 ± 0.97), respectively. The postoperative Rasmussen functional scores for the three types of fractions were 27–30 (mean 27.97 ± 0.90), 27–29 (mean 27.36 ± 0.56), and 27–29 (mean 27.56 ± 0.73), respectively.

**Conclusion:**

Flexion-valgus posterolateral tibial plateau fractures were divided into three types based on the integrity of the posterolateral wall and ACL injuries. We hope the classification can play a certain reference role in recognizing and treating flexion-valgus-type posterolateral tibial plateau fractures.

## Introduction

Tibial plateau fracture accounts for 1% of total fractures and ranks one of the most challenging fracture types for orthopedic surgeons [1]. Of all plateau fractures, posterolateral tibial plateau fractures account for 7–15% [2, 3]. This special type of fracture is caused by the axial or axial valgus stress of the knee joint in a flexion or semiflexion position, and the posterior 1/3 of the lateral tibial plateau is mainly involved [4].

In 2020, Luo et al. [5] proposed the three-column classification theory of tibial plateau fractures based on the mechanism of knee joint injury combined with CT scans. Chang et al. [6, 7] proposed the concept of a four-quadrant theory based on the understanding of isolated posterolateral fracture of the tibial plateau, dividing the tibial plateau into four parts: anterolateral, posterolateral, anteromedial, and posteromedial. Posteromedial fractures mainly manifest as split fractures with separate osteoarticular fragments of variable size. The posteromedial fragments have previously been studied radiologically, clinically, and biomechanically [8–10]. Failure of the primary fixation of the posteromedial fragment results in instability of the knee and a secondary varus deformity. However, the posterolateral fractures of the tibial plateau also need particular attention due to their more complicated anatomy. Knee instability or dysfunction will occur if the area is incompletely stabilized [11].

In 2018, we reviewed the morphology of posterolateral platform depression fractures and found that the fractures were caused by flexion-valgus injury [12]. This tibial plateau fracture pattern was classified into two subtypes on the basis of the morphological characteristics: type A was characterized by a basin-like depression of the articular surface, and type B was a tongue-like cancellous fracture resulting in a decrease in the posterior slope. The two subtypes of posterolateral platform fractures are similar in that the posterolateral cortex and the ACL are intact. Good surgical outcomes were obtained using the modified lateral approach and L-type plate fixation. Xie et al. [13] further analyzed 353 tibial plateau fractures based on both morphology and injury mechanism. They found that flexion-valgus tibial plateau fractures were mainly characterized by posterolateral articular depression, often accompanied by fracture of the posterolateral wall and/or avulsion fracture of the ACL.

Posterolateral tibial plateau fractures usually cannot be perfectly treated using traditional anterolateral or lateral approaches because of the obstruction by the fibular head and popliteus muscle [14, 15]. Lobenhoffer et al. [16] proposed a posterolateral approach using fibular osteotomy, which provided a good view of the posterolateral corner. However, this approach may lead to extensive soft tissue trauma. Therefore, Frosch et al. [2] introduced a modified surgical technique that included a lateral arthrotomy and a posterolateral approach. The ligamentous structures and soft tissues around the posterolateral fragments were protected using this approach.

We aimed to reclassify posterolateral tibial plateau fractures caused by a flexion-valgus force and describe the fracture pattern to provide a relatively programmed surgical treatment based on morphological characteristics that may improve reduction and stabilization.

## Materials and methods

### Case inclusion and exclusion criteria

**Fig. 1:**
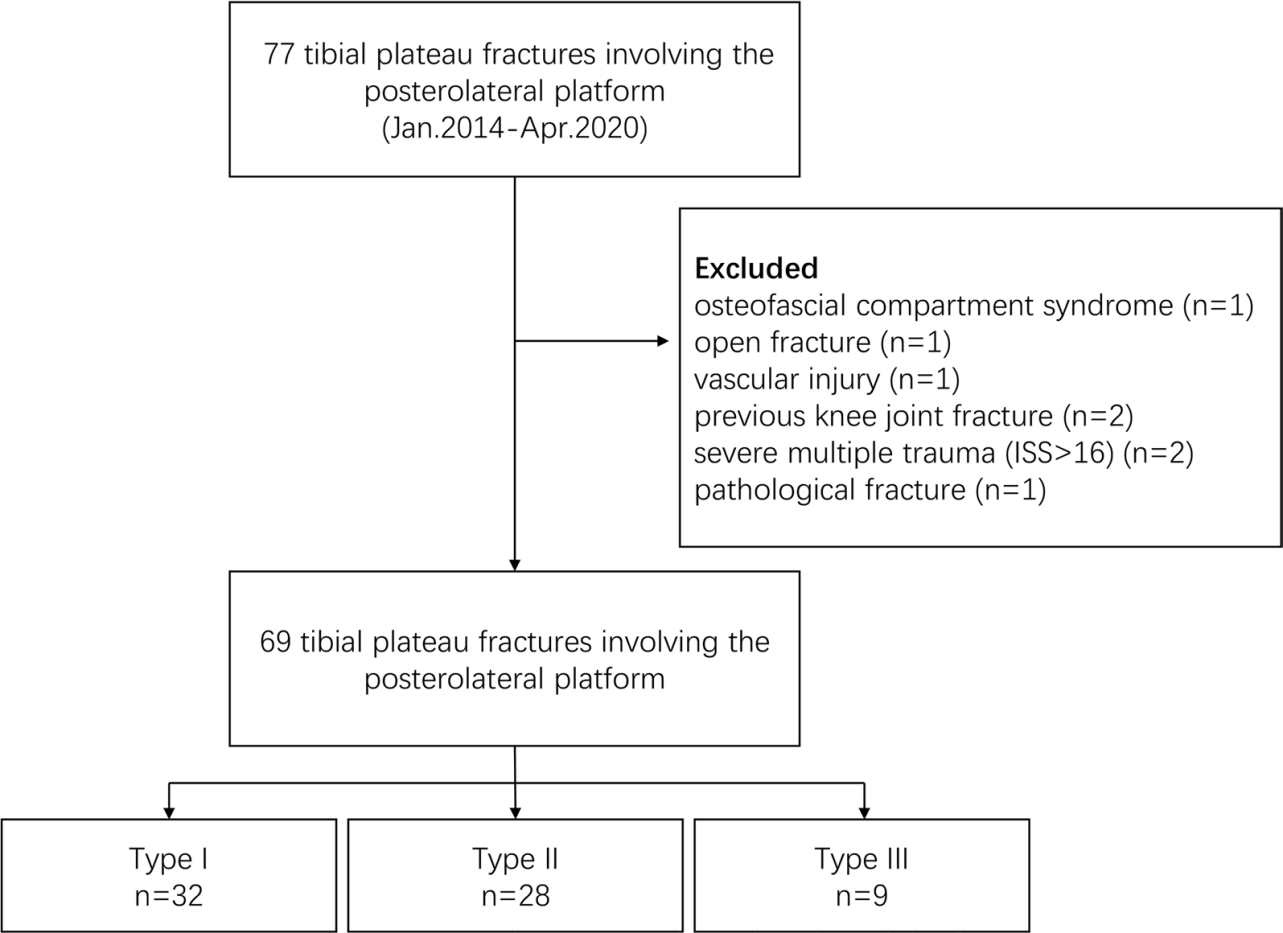
Flow-chart of the study

We retrospectively analyzed the clinical data of patients with tibial plateau fractures who underwent surgery at our hospital between January 2014 and April 2020. In total, Case inclusion criteria: (1) age > 18 years, with closed epiphysis; (2) history of trauma, closed fracture, and preoperative CT scan confirming that the lateral tibial plateau fracture involved the posterolateral condyle with posterolateral cortex fracture, with or without medial tibial plateau fracture; and (3) review of medical records confirming the mechanism of injury. Case exclusion criteria: (1) lower limb osteofascial compartment syndrome, open fracture, severe vascular and nerve injury; (2) previous knee joint fracture or knee joint deformity; (3) severe multiple trauma (trauma severity score, ISS > 16); and (4) pathological fracture (Fig. 1). This study was approved by the Ethics Committee of the First Affiliated Hospital of Nanjing Medical University.

### Surgical approach

**Fig. 2:**
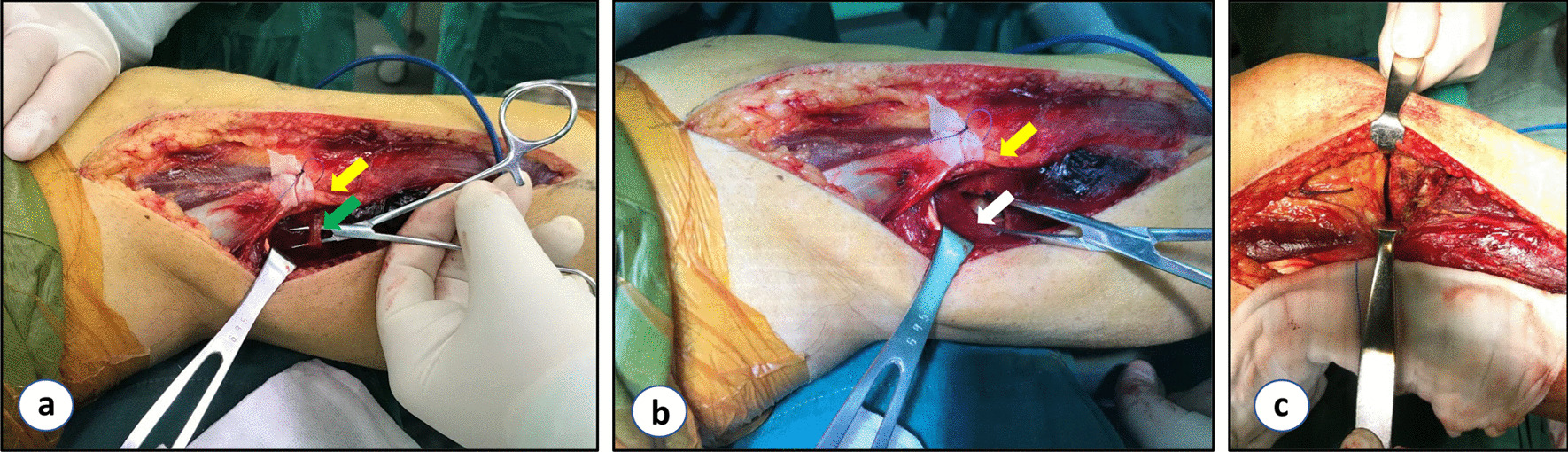
The modified Frosch approach in surgery. **a, b** Posterolateral window of the modified Frosch approach. **c** Lateral window of the modified Frosch approach. (yellow arrow: common peroneal nerve; green arrow: inferior lateral genicular artery; white arrow: popliteus.)

The patient was placed in the lateral decubitus position and the injured limb was elevated using a thick-rolled pillow. The incision was made slightly posterolateral to the knee joint and 1 cm anterior to the fibular head. It started 5 cm above the knee joint line and coursed along the anterior edge of the biceps femoris and 8 cm downward along the anterolateral side of the fibula. The tractus iliotibialis was incised from the dorsal side. The lateral capsule was then incised, and the fibular collateral ligament was retracted. The entire lateral tibial plateau, including the posterolateral corner, was visible and manipulable from this lateral window. In the posterolateral window, the common peroneal nerve was carefully dissociated and protected at the posterior edge of the biceps femoris. Blunt separation was performed between the lateral side of the gastrocnemius muscle and the posterior edge of the biceps femoris muscle. The inferior knee arteries and veins were ligated. The popliteofibular ligament was cut off and released from its fibular attachment. Then the soleus muscle was sharply separated from the dorsal surface of the fibula and drawn to the posteromedial side together with the lateral head of the gastrocnemius muscle. The posterior approach has been exposed thus far. The two windows of the modified Frosch approach were performed through one skin incision (Fig. 2).

For patients with posterolateral cortical fractures, the posterolateral fragments were first manipulated and reduced from the dorsal side through the posterolateral window. If the posterolateral platform collapsed, the articular surface was elevated and a bone graft was needed in the area with the defect. After reduction, Kirschner wires were used to fix the fragments and plain radiographs were performed using a C-arm machine to ensure the reduction. A pre-bent T-plate was used on the posterior side to buttress the fracture, and an L-plate was used on the lateral side for fixation. The meniscus was checked and repaired before closing the incision if necessary. Knee flexion exercise was started one week after surgery with the protection of a brace and weight-bearing exercise started 6–8 weeks after surgery.

### Measurements

X-rays of the knee were obtained and analyzed at 1, 3, 6, and 12 months after surgery. CT scans were obtained and analyzed at 1, 6, and 12 months after surgery. Results were assessed by three surgeons independently. The Rasmussen radiology scoring standard was used to evaluate the quality of surgical reduction. The assessment includes consideration of whether the joint surface is compressed, whether the tibial plateau has widened, and whether there is a knee varus or valgus deformity; a score of 18 is excellent, 12–17 is good, 6–11 is fair, and < 6 is poor. Knee joint function was evaluated using the Rasmussen functional score. The assessment includes subjective complaints like pain and walking capacity, as well as clinical signs like extension, total range of motion, stability; a score of 27–30 is excellent, 20–26 is good, 10–19 is fair, and < 10 is poor [17]. No patient was lost to follow-up.

### Statistical methods

SPSS statistical software (ver. 22.0 IBM Corporation, USA) was used for statistical analysis. Medial Tibial Plateau Angle (MTPA) of the affected limb was measured immediately and 3, 6, and 12 months after surgery. Pearson’s chi-square test, Kruskal–Wallis test and Fisher’s exact test were used to compare patient characteristics and scores between the groups. *p* < 0.05 was considered statistically significant.

## Results

**Fig. 3:**
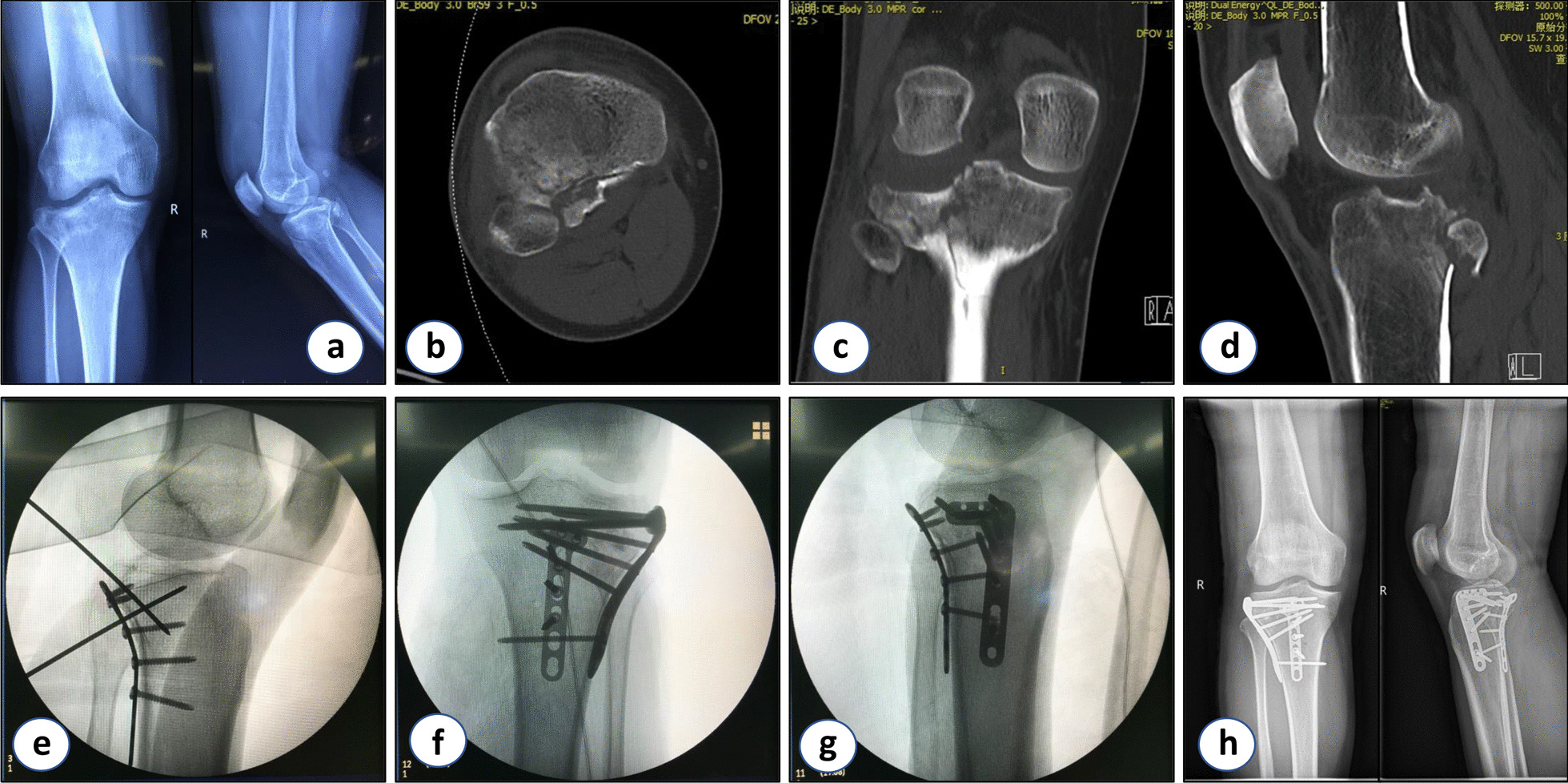
A patient with type II flexion-valgus tibial plateau fracture. **a** Preoperative X-ray examination of the knee joint. **b** Preoperative transverse CT image of the tibial plateau. **c** Preoperative coronal CT image of the tibial plateau. **d** Preoperative sagittal CT image of the tibial plateau. **e–g** Intraoperative C-arm fluoroscopy. **h** Postoperative X-ray examination of the knee joint

**Fig. 4:**
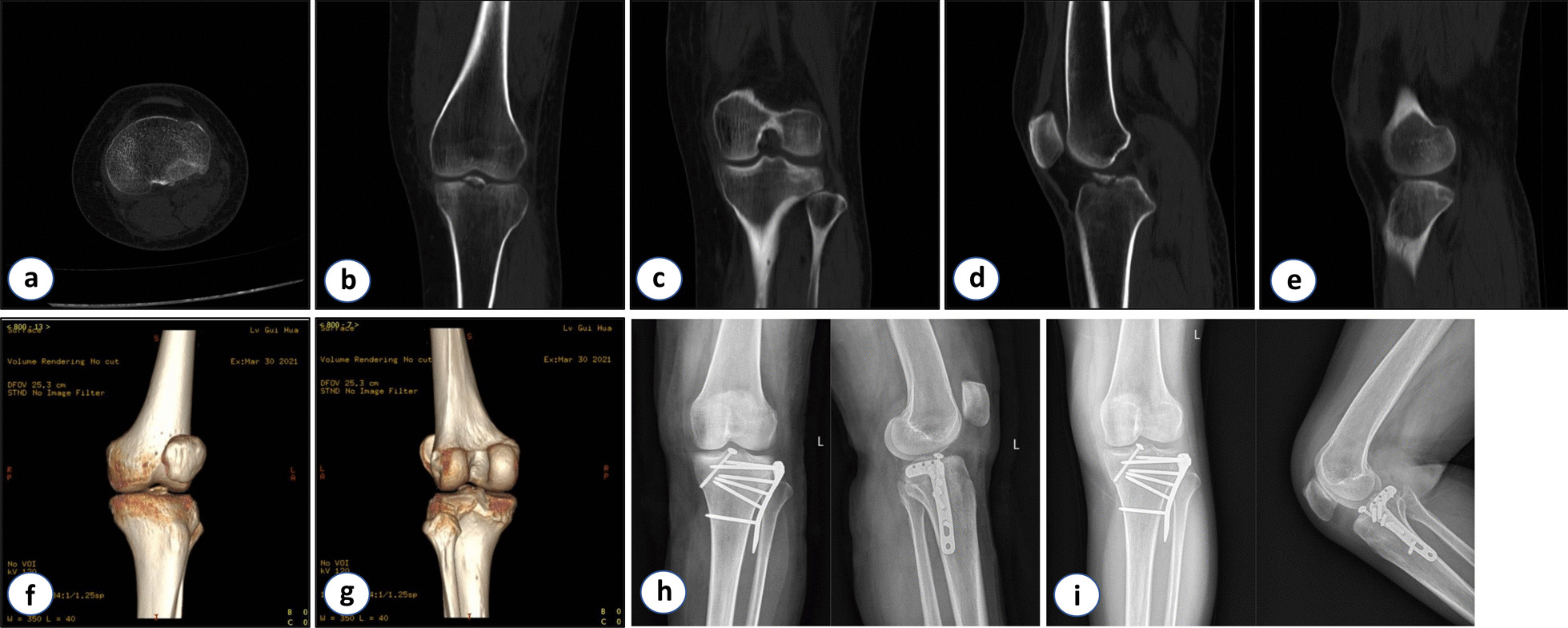
A patient with type III flexion-valgus tibial plateau fracture. **a–e** Preoperative transverse, coronal and sagittal CT images of the tibial plateau. **f**, **g** 3D reconstruction images of the tibial plateau. **h** Postoperative X-ray examination of the knee joint (3 days after surgery).** i** Postoperative X-ray examination of the knee joint (8 weeks after surgery)

A total of 69 tibial plateau fractures involving the posterolateral platform were identified and included in this study (Table 1). Based on the AO/OTA classification, 41 type 41B2 fractures and 28 type 41B3 fractures were identified. Through medical record review, we found that all patients reported flexion-valgus force at the moment of the accident. Based on the fracture morphology in the preoperative CT scans, the cohort was divided into three types. Type I was a depression fracture of the posterolateral platform with intact posterolateral cortex (32 cases). Type II was a depression fracture of the posterolateral platform with fractured posterolateral cortex (28 cases). Type III was a depression fracture of the posterolateral platform in combination with ACL rupture or tibial insertion avulsion fracture of the ACL (9 cases) (Table 2). For patients with type I and type III posterolateral tibial plateau fractures, the lateral window of the modified Frosch approach and an L-type locking plate were used, as previously reported. For patients with type II posterolateral tibial plateau fractures, both the lateral and posterolateral windows of the modified Frosch double-space approach were used for surgery, and a T-plate on the posterior side together with an L-plate on the anterolateral side were used for fixation. The final reduction and implant placement were confirmed with intra-operative radiographs prior to closure. All fractures were well reduced and the articular surface steps were less than 2 mm. All screws were placed below the articular surface (Figs. 3 and 4).

**Table 1:** Patient demographics

Subtypes of fractures	I	II	III	Total	*p* value
Age (y)	43.37 ± 10.91 (25–68)	44.96 ± 9.18 (29–64)	40.44 ± 9.90 (29–57)	–	0.174
Gender					0.096
Male	14	16	6	36	
Female	18	12	3	33	
Side of injury					0.077
Left	12	13	5	30	
Right	20	15	4	39	
Operation time (min)	96.72 ± 9.89 (85–130)	132.32 ± 17.82 (105–165)	109.44 ± 9.82 (95–125)		**0.005**
Blood loss (ml)	157.81 ± 47.70 (100–250)	373.21 ± 97.64 (200–650)	227.78 ± 66.67 (100–300)	–	**0.003**
Rasmussen radiological scores	16.31 ± 0.78 (15–17)/Good	15.93 ± 0.94 (14–17)/Good	16.22 ± 0.97 (14–17)/Good	–	0.051
Rasmussen functional scores	27.97 ± 0.90 (27–30)/Excellent	27.36 ± 0.56 (27–29)/Excellent	27.56 ± 0.73 (27–29)/Excellent	–	0.104

**Table 2:** The classification of the flexion valgus fracture

Subtypes	Configuration of the fracture	Status of the column cortex	Status of the ACL	Injury mechanism	Surgical approaches for fractures
I	Depression fracture of the posterolateral platform	Intact	Intact	Flexion-valgus force	Lateral window of Frosch approach
II	Split and depression fracture of the posterolateral platform	Incomplete	Intact	Flexion-valgus force	Lateral and posterolateral windows of Frosch approaches
III	Depression fracture of the posterolateral platform	Intact	Ruptured or tibial insertion avulsion fracture of ACL	Flexion-valgus force and internal rotation of tibia	Percutaneously or lateral window of Frosch approach

The operation time for the three types of fractures was 85–130 (mean 96.72 ± 9.89) min, 105–165 (mean 132.32 ± 17.82) min, and 95–125 (mean 109.44 ± 9.82) min, respectively. The blood loss during surgery were 100–250 (mean 157.81 ± 47.70) ml, 200–650 (mean 373.21 ± 97.64) ml, and 100–300 (mean 227.78 ± 66.67) ml, respectively. All patients were followed up for 12–30 (mean 20.8 ± 9.4) months. No blood vessel injury occurred during the surgery. No deep infections or skin necrosis occurred after surgery. One patient with of type II fracture in this study had symptoms of common peroneal nerve injury after surgery but recovered 6 months later. All fractures healed, and the healing time assessed by X-ray was 12–23 (mean 15.6 ± 5.2) weeks. There was no joint surface subsidence, reduction loss, or screw mal-positioning. No sagittal instability of the knee joint was found, and the drawer test was negative in all patients 3 months after surgery.

The Rasmussen radiological scores ranged from 14 to17 points. The three types of fractures had the following results: type I: 15–17 (mean 16.31 ± 0.78); type II: 14–17 (mean 15.93 ± 0.94); and type III: 14–17 (mean 16.22 ± 0.97). There was no significant discrepancy among the groups (*p* = 0.572). The results were “good” as interpreted by Rasmussen radiological scores with points ranging from 14 to 17. The Rasmussen functional scores ranged from 27–30 points. The three types of fractures showed the following results: type I: 27–30 (mean 27.97 ± 0.90); type II: 27–29 (mean 27.36 ± 0.56); type III: 27–29 (mean 27.56 ± 0.73). There was no significant discrepancy among the groups (*p* = 0.867). The results were “excellent”, as interpreted by Rasmussen functional scores, with points ranging from 27 to 30 (Table 1).

## Discussion

The most important finding of the study was that we divided flexion-valgus posterolateral tibial plateau fractures into three types according to the integrity of the posterolateral wall and ACL injury. The posterolateral tibial plateau fractures are special types of fractures that are relatively rare in clinical practice. Vertical force such as traffic accidents and fall injuries is the most common cause. Through medical record review, we found that the posterolateral tibial plateau fractures were caused by flexion-valgus force. Flexion-valgus force caused by traffic accidents accounted for almost 70% of all injuries, of which the vast majority were caused by electric vehicles. When an accident occurred, the knee of the electric vehicle driver suffered axial and valgus trauma in the flexed position, and the posterolateral tibial platform was hit by the lateral femoral condyle. This compression might lead to posterolateral fractures, and most of the fractures are isolated posterolateral collapses of the tibial plateau or cleavage collapses [18, 19]. The energy that causes posterolateral tibial plateau fracture with an intact cortex is less than that which causes posterolateral tibial plateau fracture with a disrupted cortex. This could be clearly observed from the faster speed and the larger size of the electric vehicles involved in the accident. Additionally, disruption of the posterolateral cortex is closely related to the knee joint movement trend and the angle of knee flexion at the moment of injury because the center of the femoral condyle moves backward during knee flexion [20, 21]. According to Speer et al. [22], ACL injuries indicate that the knees experience subluxation and internal rotation during the accident. Therefore, posterolateral platform fractures with ACL injuries indicate that flexion-valgus force occurs when knee subluxation and internal tibial rotation occur [23].

Researchers have proposed many classifications for tibial plateau fractures based on the fracture morphology or injury mechanism. However, no classifications that specifically target the posterolateral platform of the tibial plateau is universally accepted and utilized. In this study, the flexion-valgus posterolateral tibial plateau fractures were divided into three types based on fracture morphology, injury mechanism, and with a view of treatment. Type I fractures were depression fractures, and the posterolateral cortex was intact. We previously described this type of fracture and further divided it into type IA and type IB fractures, namely basin-like fractures and tongue-like fractures [12]. For basin-like depression fractures, surgeries are always needed when the articular surface step exceeds 3 mm for because long-term traumatic arthritis may develop due to stress concentration around the fracture site. Physical examination of the knee joints are required for tongue-like depression fractures, and surgeries are only considered when the depression exceeds 3 mm and the knee joints are unstable. The integrity of the posterolateral wall is broken in patients with type II fractures. Selection of surgical approaches and fixation methods are more difficult in these fractures than in those without a broken posterolateral cortex. Type III fractures are posterolateral fractures with ACL injury or tibial insertion avulsion fracture of the ACL. Although the fragment depression of the posterolateral platform may be small, regarded as clinically irrelevant, or even missed, attention is required in posterolateral tibial plateau fractures with ACL injury or tibial insertion avulsion fracture of ACL, the so-called apple-bite fractures [24]. Rotatory instability of the knee joint may persist if not well treated. A posterolateral tibial plateau fracture with a depression greater than 2 mm with an ACL injury or ACL tibial insertion avulsion fracture and a grossly positive pivot shift test should be properly treated to avoid persistent knee instability [25, 26].

The ideal surgical approach should provide a sufficient field for vision and surgery, and minimize soft tissue damage, infection, and postoperative complications. Many surgical approaches are used to treat the posterolateral tibial plateau in clinical practice, which can be mainly divided into anterolateral approaches, posterolateral approaches, and osteotomy approaches [27]. It is difficult to directly expose the posterolateral tibial fractures due to obstruction by the fibular head and common peroneal nerve. To address this issue, Lobenhoffer et al. [16] developed a posterolateral approach in 1997. Using this approach, fibular osteotomy was performed, and a good view of the posterolateral joint surface of the tibial plateau was obtained. However, considerable trauma to the soft tissue of the posterolateral corner was caused by this approach. Luo et al. [28] proposed the use of an anterolateral combined with a posteromedial inverted L-shaped incision to treat tibial plateau fractures in 2012, which exhibited certain advantages in treating combined posterolateral and posteromedial fractures. However, excessive stretching of the gastrocnemius muscle during intraoperative reduction and fixation may increase the damage to nerves, vascular bundles, and muscle soft tissues behind the knee joint [29]. Moreover, the medial head of the gastrocnemius sometimes needs to be cut off to achieve better surgical vision, which is not conducive to early functional exercise of the knee joint [30]. Frosch et al. [2] introduced a modified surgical technique that included a lateral arthrotomy and a posterolateral approach. The ligamentous structures and soft tissues around the posterolateral fragments were protected using this approach. In 2018, we published a new strategy to treat posterolateral compressive tibial plateau fractures using this novel lateral approach [12], namely, the lateral window of the modified Frosch approach. The approach could provide excellent visibility and surgical access to the posterolateral quadrant of the tibial plateau without the need for complex deep dissection or osteotomy, and eliminates the risk of damage to trifurcation vessels or the posterolateral ligament complex. However, it is only suitable for posterolateral tibial plateau fractures with an intact posterolateral cortex, namely type I and type III posterolateral fractures. For patients with posterolateral cortical comminuted fractures, reduction from the anterolateral side may aggravate posterior displacement of the fracture. Therefore, both lateral and posterolateral windows of the modified Frosch approach were used for type II posterolateral tibial plateau fractures. The approach provides good vision and operation space for the entire lateral platform from the lateral side and rear. It does not require osteotomy of the fibular head, which protects the stability of the posterior structure of the knee joint. With the advantages of the modified Frosch approach, posterolateral cortex fractures can first be fixed and supported from the rear.

The fixation devices specifically designed for posterolateral platform fractures remain vacant for a long time. Some scholars have recently designed an anatomical locking plate for the posterolateral fractures of the tibial plateau, which is expected to be gradually applied in the future [31]. Currently, distal radius T-type plates or reconstruction plates are often used in clinical practice. A study showed that the anterior tibial artery was 27–62 mm (mean 46.3 ± 9.0 mm) from the tibial plateau [32]. Therefore, to avoid vessel damage, the operation should not exceed 4 cm below the posterolateral platform. Subsequently, the fractures were reduced and fixed from the anterolateral side. The 3.5 mm L-type locking anatomical plate on the lateral side of the proximal tibia selected in this study had a proximal width of 10 mm and a thickness of 3 mm and was designed with 4 locking holds for rafting screws. These features make it a good option for posterolateral tibial plateau fractures. A biomechanical study performed by Karunakar et al. [33] showed that the use of a locking plate with 4 rafting screws provided significantly better local compression stiffness than the use of a supporting plate combined with bone grafting.

Full weight-bearing is prohibited for 6 weeks after surgery, but early functional exercise of knee extension and flexion in a non-weight-bearing state is allowed. The lateral femoral condyle gradually rolls back and translates during knee flexion, and the posterolateral platform can conduct stress only when the knee flexes > 90° [34, 35]. Therefore, non-weight-bearing functional exercise does not result in a large axial compression shear force on the posterolateral fragments.

## Limitations

This study had some limitations. First, the sample size was small owing to the low incidence of injuries, especially type III fractures. A larger sample size may provide more valuable insights. Second, we mainly focused on the bone structure of the tibial plateau while neglecting the soft tissues around the knee joint, which also play important roles in tibial plateau fractures and knee stability. Third, this is a newly proposed classification focused on flexion-valgus posterolateral tibial plateau fractures, which means that the reliability of the classification and the prognostic value remain to be evaluated.

## Conclusion

According to the integrity of the posterolateral wall and ACL injury, flexion-valgus posterolateral tibial plateau fractures were divided into three types. We hope that this classification can play a certain reference role in the recognition and treatment of flexion valgus tibial plateau fractures.
